# Emergence, Reduction and the Identity and Individuation of Powers

**DOI:** 10.1007/s11245-018-9621-x

**Published:** 2018-12-10

**Authors:** Alexander Daniel Carruth

**Affiliations:** Department of Philosophy, Durham University, 50 Old Elvet, Durham, DH1 3HN UK

**Keywords:** Emergence, Reduction, Powers, Single-track, Multi-track, Stimulus, Manifestation, Mutual manifestation

## Abstract

One recently popular way to characterise strong emergence is to say that emergent entities possess *novel causal powers*. However, there is little agreement concerning the nature of powers. One controversy involves whether powers are single- or multi-track; that is, whether each power has only one manifestation type, or whether a single power can be directed towards a number of distinct manifestations. Another concerns *how* powers operate: whether a lone power manifests when triggered by the presence of a suitable stimulus, or whether powers operate mutually such that several powers must ‘work together’ to bring about a particular manifestation. This paper examines how these distinctions—which can be cross-combined to frame four distinct accounts of the nature of powers—bear on the debate between emergentists and reductionists.

## Introduction

Emergentists hold that higher-level phenomena, such as the mind, are something ‘over and above’ the sum of their most basic parts. This usually involves the emergent phenomena being taken to be both *distinct from* and *novel with respect to* the base phenomena from which they emerge, whilst nevertheless being dependent upon the base phenomena. How *distinctness* and *novelty* should be understood depends on the kind of emergence being proposed: epistemically emergent higher-level phenomena are indispensable features of certain explanatory or predictive practices; whereas with metaphysically emergent phenomena their ‘over and above-ness’ is a matter of ontology. This division of kinds of emergence into epistemic and metaphysical is neither exhaustive nor maximally specific, but it should be sufficient for the purposes of this paper. For those interested in a more nuanced division, there has been a lot of recent work on varieties of emergence, see for instance: Chalmers (2006), Silberstein (2001), van Gulick (2001) and Wilson (2015).

This paper focusses on the *ontological* debate between emergentists and reductionists, with special attention paid to the thesis that the *mind* is a strongly emergent entity. To hold that the mental reduces to the neurological or physical *ontologically* involves commitment to the claim that the mind is *nothing over and above* some underlying physical entity such as the brain—*prima facie* distinctively mental phenomena such as thoughts, emotions and experiences are fully constituted by or identical with purely physical phenomena such as electro-chemical brain processes.

One popular way to characterise a strong form of metaphysical emergence is to say that emergent entities must possess *novel causal powers*. For instance, Jaegwon Kim asserts that if emergentism is to be a coherent position, then emergent entities must have distinctive causal powers (1999). Timothy O’Connor and Hong Yu Wong characterise emergent properties as basic properties had by composite entities; where ‘basicness’ is at least in part a matter of conferring novel causal powers (2005). Jessica Wilson takes strongly metaphysically emergent entities to have “fundamentally novel powers” (2015, p. 356). According to these sorts of view, a strongly emergent mind would have to be poised to make a distinctive causal contribution to the world, one which exceeds the causal potential of the base entities from which it emerges. Whilst this powers-based approach to strong emergence is not the only possible view, the discussion in this paper will be restricted to a conception of emergence whereby for some entity E to be an emergent entity, E must have causal powers which are not had by the base entities, the Bs, upon which E depends.

## Why Understand Emergence in Terms of Causal Powers?

There are a number of compelling reasons to frame strong metaphysical emergence in terms of the possession of distinct, novel causal powers. First, suppose, for the sake of argument, that E is a higher-level object which is dependent on some set of lower-level objects, the Bs, but that E possesses causal powers which are genuinely distinct from and novel with respect to the causal powers possessed by the Bs. Remember that the issue at stake, in terms of strong metaphysical emergence, is whether there exist any higher-level entities which are properly characterised as ‘over-and-above’ the lower level entities upon which they depend. E’s having its own causal efficacy which cannot be attributed to the Bs looks like evidence *par excellence* that E is something over and above the Bs; that E ought to be accorded genuine irreducible ontological status. If it were not, then it seems that there would be no entity to which to attribute this causal efficacy, and this seems absurd.

Second, having a distinctive causal role seems the appropriate criterion for picking out *strong* forms of metaphysical emergence. Suppose that there could be grounds for thinking that some higher-level entity E_1_ was in some sense metaphysically, and not merely epistemically—that is, merely with regards to explanatory or predictive practices—distinct from and novel with respect to the lower-level entities, the Bs, upon which it depends. E_1_ might well count as metaphysically emergent, then. But compare E_1_ to a higher-level entity E_2_ which *does* have its own novel causal powers—it seems very natural to see the latter as emergent in a *stronger* sense than the former. This kind of distinction between strong and weak metaphysical emergence can be found, for instance, in Wilson (2015).

Third, to the extent to which one has confidence in science’s ability to trace causal goings-on, adopting a powers-based conception of strong emergence promises to help make the debate between emergentists and reductionists empirically tractable—at least in part.^1^ This is something that all parties to the debate ought to agree is desirable. For arguments in favour of the claim that one of the central roles of science, especially the physical sciences, is to trace causal goings-on, see for instance, Blackburn (1990) or Hawthorne (2001).

Fourth, adopting a power-based conception of emergence will not only be attractive to emergentists, as its combination with various forms of causal exclusion argument (see e.g. Kim 1999) provides a clear framework and procedure for reductionism [this point is nicely elaborated in Elder (2003)]. Having a conception that both emergentists and reductionists can agree upon is crucial for the debate. If Smith is an emergentist concerning entities in some domain, but Jones is reductionist about them, and Smith and Jones subscribe to differing conceptions of emergence, then there is a very strong chance that there is only the appearance of genuine disagreement between them—Smith and Jones may well simply be talking past one another. The form of reduction that stems from the acceptance of a powers-based conception might also be thought attractive because it is clear to see how it can be distinguished from eliminativism—if E reduces to the Bs, as all of E’s causal powers can be identified with causal powers of the Bs, then according to this view E still *exists*—we have just been ascribing causal powers to E! E does not, however, exist as *distinct from* or *novel with respect to* the Bs: E is nothing *over and above* the Bs.

See Carruth (forthcoming) for further discussion of causal powers in the emergence debate, especially regarding the role played by ‘Alexander’s dictum’ in this context.

## Causal Powers

Causal powers are features or properties of objects in virtue of which the objects that instantiate them causally interact in the manner that they do.^2^ Objects with just the same causal powers will behave in the same way when placed in exactly similar circumstances. Objects with different causal powers will behave differently, and this difference in behaviour will be due to their instantiating different causal powers. Views such as strong versions of dispositional essentialism hold that all fundamental properties are causal powers (see Shoemaker 1980). Others take causal powers to depend on or reduce to a combination of non-causal properties and the laws of nature (e.g. Armstrong 1997). Others still take some fundamental properties to be powers, and others no to be (e.g. Molnar 2003). This paper will not aim to settle this dispute. However, it should be noted that the various approaches to the nature of powers to be discussed in this paper have been developed within frameworks which adopt a robust, metaphysically serious attitude towards powers—those approaching the issue from the perspective of neo-Humean metaphysics may be less likely to be attracted to the powers-based conception of emergence, and less amenable to the various views on the nature of powers set out in section four.

Causal powers are essentially powers *to* something or other. That is, their nature involves being directed towards some manifestation or set of manifestations; and they bring about these manifestations in suitable circumstances. Canonical examples of causal powers include for instance fragility, which could be roughly characterised as ‘the power to break when struck’, or solubility, ‘the power to dissolve when in contact with a suitable solvent’. We can roughly capture these features using conditional statements of the form:

### CP

x has the power to φ if it is the case that were x to be placed in suitable circumstances C, then x would φ.

I don’t offer **CP** as an *analysis* of what it is to have a power, although some philosophers have attempted to analyse powers in terms of the truth of conditional statements [e.g Lewis (1997); such analyses are plagued by familiar counterexamples, however, see Martin (1994) and Bird (1998)]. But conditional statements such as **CP**, even if they fail to analyse what it is to have a power, capture at the central features of powers mentioned above: their directedness and their sensitivity to circumstance.

There are several controversies in the debate concerning the nature of powers. One concerns whether causal powers are ‘single-’ or ‘multi-track’; that is, whether a given power only ever disposes its bearer towards a single manifestation or whether an object can be disposed towards a range of manifestations just in virtue of a single power it instantiates. Another controversy concerns the way powers operate. One view has it that this involves the power being ‘triggered’ by some stimulus, which leads to the production of the manifestation. The other holds that when a power produces its manifestation, this always involves mutual action with other powers.

Single-track powers have only a single manifestation, or perhaps a single manifestation type. Whenever a single-track power manifests, and in whatever circumstances it does so, the manner in which it manifests is the same. Conversely, multi-track powers are directed towards a range of different manifestations, and so on two different occasions, when such a power manifests, it may manifest in different ways, depending on the circumstances in question. There are at least two ways in which a power might be multi-track. Neil Williams (2011) discusses cases of *quantitative* multi-tracking, where a single power is directed towards a range of manifestations which are similar but differ with regards to degree: for instance, consider the *elasticity* of a particular rubber band. This power might be conceived as the power to undergo deformation followed by a return to original shape within a given range: you can stretch the rubber band by two, three or five centimetres, but stretch it too far and it will snap. The thought is that all these various manifestations should be attributed to just the one power: it would be wrong, having observed the various deformations the band can undergo, to hold that it had a group of distinct powers such as *the-power-to-stretch-by-two-centimetres, the-power-to-stretch-by-three-centimetres* and so on.

Vetter (2013) and Heil (2003) have both discussed cases of *qualitative* multi-tracking. In these cases, unlike the quantitative cases, a single power can be directed towards a range of manifestations not just of differing degree, but which differ by type. Heil writes:

“Consider a simple case, the sphericity of a particular ball. The ball’s sphericity, in concert with incoming light radiation, structures outgoing radiation in a definite way. The very same property of the ball disposes it to produce a concave depression in a lump of clay or to roll... one disposition, many different kinds of manifestation.” (2003, pp. 198–199).

Both quantitative and qualitative conceptions of multi-tracking allow that a given power might have a relatively restricted number of tracks, or might have a very large, maybe even infinite, number of tracks—Martin (2008) holds the latter view. Some proponents of single-tracking, for instance Lowe (2010), have argued that quantitative multi-tracking should not be accepted as ‘genuine’ multi-tracking, as the various tracks can be subsumed under a single manifestation-type and unifying description. Quantitative multi-trackers, for instance Williams (2011), have however provided counter-arguments to this claim. It should be clear that whether powers are single- or multi-track has important ramifications for how we identify and individuate powers, both theoretically and empirically: at least under certain conditions, the two camps disagree both over the number of powers that a candidate object instantiates, and with regards to what constitutes evidence in favour of attributing a power to an object.

Another issue concerning the nature of powers involves considering how it is that they come to manifest, that is, how they operate. Different accounts of how powers operate are no less important to determining the identity of a power than is the manifestation(s) towards which the power is directed.

One account of how powers operate is the ‘stimulus-manifestation’ model, which is arguably the orthodox position in the contemporary literature (e.g Bird 2007). According to this account, a causal power will only give rise to a manifestation when it is galvanised into action by some trigger or stimulus. For instance, in the case of the *fragility* of a vase, the stimulus might be ‘being struck with a force greater than X’, or in the case of the *solubility* of a sample of salt, ‘being submerged in water’. The manifestation is produced by the target power alone, although it will not be produced until the occurrence of the stimulus. Thus, on this account a power’s nature could be specified in terms of a relationship between the power, its manifestation or manifestations and a stimulus or some stimuli. Tugby (2010) has argued that stimuli needn’t themselves be powers, and that there may be a certain heterogeneity with regard to the nature of stimuli. For instance, stimuli may be actions such as ‘being struck with a force greater than X’; or they may be states such as ‘being submerged in water’.

An alternative account is the ‘mutual manifestation’ model. According to this view, in order for some manifestation to occur, there must always be at least two powers working together to bring it about. When they do so, there is no sense of priority such that one power could be considered the operative power, whilst the other is held to have merely stimulated or triggered it. For instance, in the case of the production of a particular vase’s shattering, this view would hold that this is not the result of the ‘fragility’ of the vase alone, but rather of a whole host of causal powers of the vase, of the object that struck it, and perhaps more besides. Likewise, the dissolution of a sample of salt is a result of the mutual action of both the particular crystalline structure of the salt and the dipole moment typical of H_2_O molecules (and perhaps more besides). Thus, on this account a power’s nature could be specified in terms of a relationship between the power, its manifestation or manifestations and the partner or partners with which the power mutually manifests.

The mutual manifestation view allows that powers operate in a pair-wise manner and also that higher-adicity interactions are possible. Indeed, Tugby (2013) argues that there are both empirical and metaphysical reasons to support the claim that those who hold a mutual manifestation view should allow for interactions involving more than two powers. This account of the manner in which powers operate is defended, amongst others, by Martin (2008), Heil (2003), Mumford and Anjum (2011) and Tugby (2013).

Both debates discussed so far—that is, the tracking and operation of powers—have ramifications for how powers are identified and individuated. Thus, anyone hoping to deploy the notion of powers should take into account how their resolution might impact upon the given context in which the notion is used—in this case, on the notion of strong emergence.

## Four Accounts of Causal Powers

The distinctions between single- and multi-tracking, and between the stimulus-manifestation and mutual manifestation views can be cross-combined to frame four distinct accounts of causal powers. This paper will not address arguments for or against these four accounts, although it will be indicated where and by whom each account, or at least something like them, are defended or discussed in the literature.

**Fig. 1:**
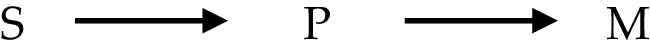
Schematic diagram representing the stimulus-power-manifestation relationship

In what follows the four views of powers will be illustrated partially by use of diagrams, and so a quick note is required to explain how these are to be interpreted. The diagrams consist of letters standing for various phenomena, which are labelled by use of numbers in subscript; and of arrows connecting the letters. In each diagram, ‘S’ stands for ‘stimulus’; ‘P’ stands for ‘power’, and ‘M’ stands for ‘manifestation’. Sameness of subscript implies type-identity/exact similarity; difference of subscript implies type-non-identity/lack of exact similarity: for instance, where ‘S_1_’ appears in two different diagrams, it picks out two instances of the same stimulus, say ‘being struck with a force greater than X’, and where ‘S_1_’ and ‘S_2_’ appear either in the same or different diagrams, they pick out instances of different stimuli, say ‘being struck with a force greater than X’ and ‘being submerged in water’ respectively. The arrows are a placeholder for the obtaining of some relationship or other the precise nature of which needn’t detain us here and may not pick out the same relationship every time they appear even within a diagram. Thus, it might be natural to read the following (Fig. 1): as, ‘S *stimulates* P *to produce* M’, where the text in italics picks out the relevant relationships. Where multi-track powers are represented, different styles of line such as solid, dotted or dashed, indicate which stimulus/pairing produces which manifestation.

### Single-Track Stimulus Manifestation

**Fig. 2:**
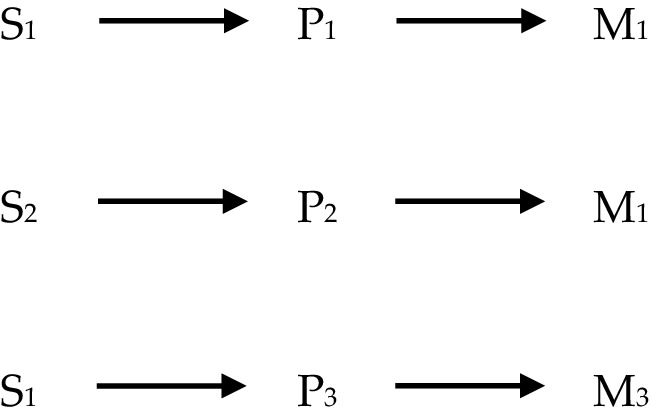
Single-track stimulus manifestation powers

On this view, the identity of a power can be specified as a triadic relationship between a single stimulus; the power, and a single manifestation, as depicted in Fig. 2.

One consequence of such an account is that powers are individuated both by their specific stimulus and the specific manifestation towards which they are directed. Thus, the very same manifestation, if it occurs following distinct stimuli, must be brought about by a distinct power (as in the top two lines of Fig. 2). Likewise, the very same stimulus can lead to different manifestations when it triggers different powers (as in the top and bottom lines of Fig. 2). This way of thinking about powers aligns most closely with **CP**, introduced earlier, and is arguably the orthodox conception of causal powers. It is defended by Bird (2007) amongst others.

### Multi-track Stimulus Manifestation

One can hold that the powers operate on the stimulus-manifestation model and reject single-tracking. For instance, Williams says:

“Powers [directed towards multiple manifestations] would be single powers that respond differently for different stimuli—and that just is what it is to be a multi-track power.” (2011, p. 590)

**Fig. 3:**
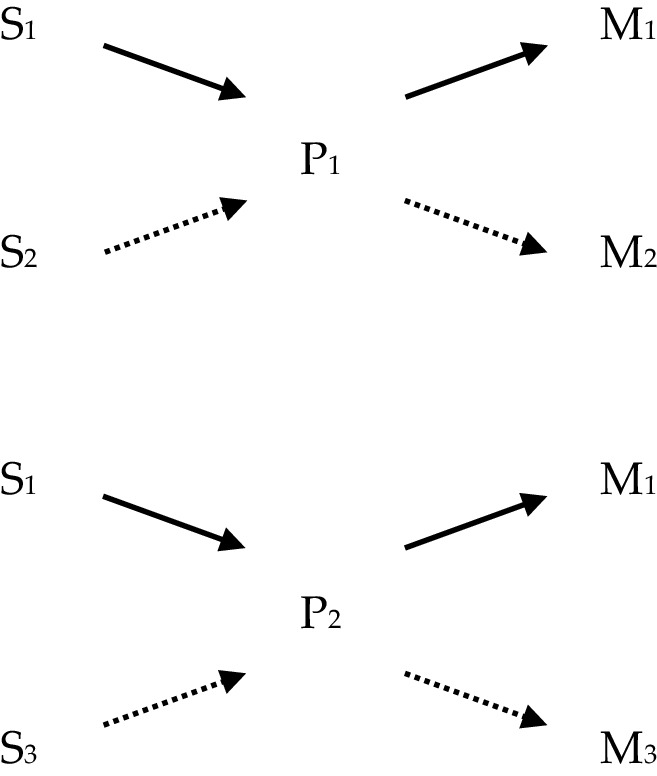
Multi-track stimulus manifestation powers

On this view the identity of a power at least partly consists in an n-adic relationship between the power, a set of stimuli and a set of manifestations. This view has the interesting consequence that the very same manifestation can occur on two occasions following the very same stimuli but be due to a different power in each case (as depicted in Fig. 3 when looking at stimulus-power-manifestation triads indicated with solid-line arrows in each diagram). Two distinct powers thus may manifest in the same way in a variety of situations, but differently in others.

There are interesting ramifications of this view for the epistemology of powers: two distinct powers may overlap with regard to almost all their ‘tracks’, and all the stimuli that they typically encounter might produce the very same manifestations. Nevertheless, there may be some highly atypical stimuli which would differentiate them, although in fact we are rarely or never in a position to observe the manifestation brought about by this atypical, differentiating stimulus.

### Single-Track Mutual Manifestation

Another option is that powers are single-track but operate according to the mutual manifestation model. Thus, the identity of a given power at least partly consists in a relationship between the power, a manifestation and at least one other ‘partner’ power. On this view, because powers are held to be single-track, it cannot be the case that the very same power can produce different manifestations with different partners.

**Fig. 4:**
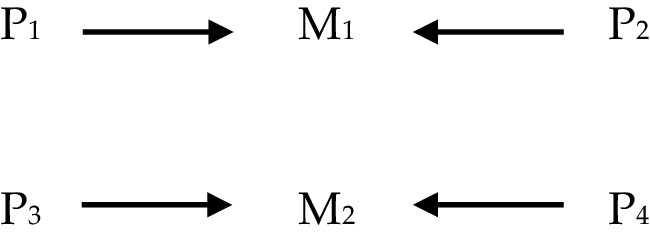
Single-track mutual manifestation powers

Whilst the other views in this section find explicit defenders in the literature, I am not aware of anyone who self-consciously endorses the single-track mutual manifestation model. However, something like this view is perhaps that held by those who distinguish between ‘active’ and ‘passive’ powers or causal powers and causal liabilities (e.g. Lowe 2013), where in order for some manifestation to occur—say salt’s dissolving in water—the water must have the ‘active’ power to dissolve and the salt the ‘passive’ power or liability to be dissolved. On such a view, there is a single typifying manifestation, but multiple powers are required for its production (Fig. 4).

A couple of points to note: first, few supporters of mutual manifestation also endorse the active/passive distinction—the idea is not to run these two views together, but to attempt to provide an example of a specifically *single-track* mutual manifestation view; second, it is probably possible to combine the active/passive distinction with multi-tracking, but the way the view is most typically expressed seems to align it more with a single-track approach.

### Multi-track Mutual Manifestation

The final account combines the mutual manifestation model of how powers operate with the view that powers are multi-track. On such a view, the identity of a power could be specified in terms of an n-adic relationship between the power, a set of manifestations and a set of mutual partners. This kind of view might suggest a holism about the identity of powers, for instance, see Martin (2008).

**Fig. 5:**
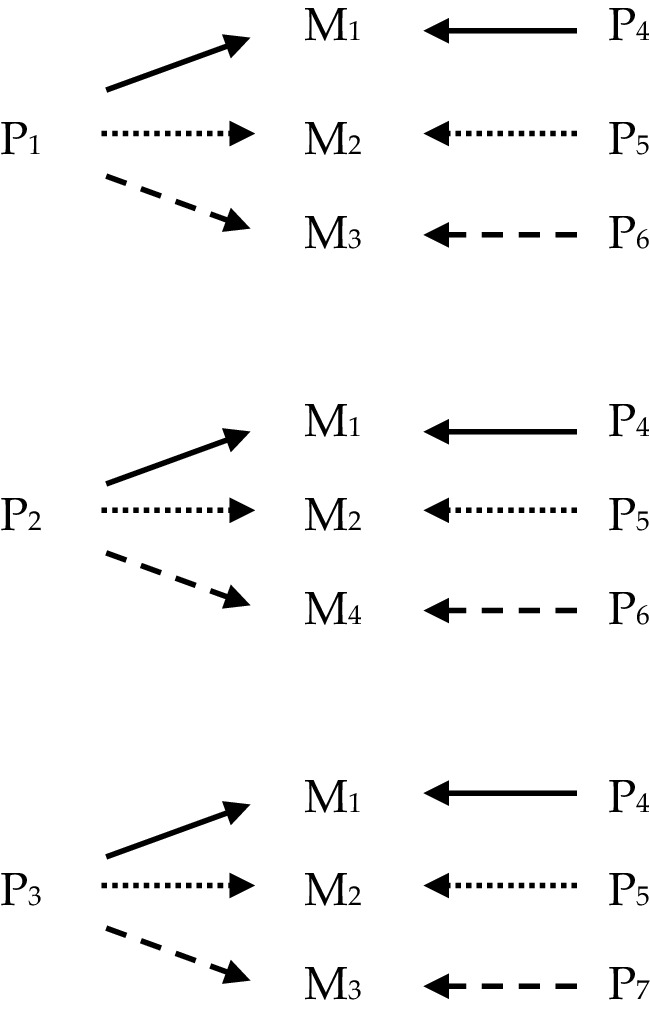
Multi-track mutual manifestation powers

Thus, two distinct powers may manifest in the same way in a variety of situations, but differently in others—and indeed may share almost all their typical mutual partners, as is the case with P_1_ and P_2_ in Fig. 5, which has similar consequences for the epistemology of powers as those mentioned in Sect. 4.2 when discussing the *multi-track stimulus manifestation* view. Likewise, this view has the consequence that two distinct powers may share all their manifestations, but be genuinely distinct because some or all of them are brought about with different partners, as is the case with P_1_ and P_3_ in Fig. 5.

This sort of view is advanced in Martin (2008) and Heil (2003).

Having outlined these four different approaches to the identity and individuation of causal powers, we are now in a position to examine how adopting each approach might bear on the debate between concerning strong emergence, when emergence is cashed out in terms of novel causal powers.

## Theories of Causal Powers and the Emergence–Reduction Debate

Consider a dialectic which is common in the debate between emergentists and reductionists: first, some distinctive form of behaviour or phenomena which only occurs in certain complex situations is identified which, *prima facie*, is candidate evidence of emergence. In the case of the mind, the manifestation of experiential qualities or intentional states could be considered examples of this kind: as far as we know, it is only when certain highly specific and complex conditions are met—those required to sustain a functioning brain in a living body—that these phenomena occur. It is then open for debate whether or not the powers which are usually attributed to the relatively simple components of this complex situation are sufficient to account for the behaviour or phenomena—in the case of the mind, whether neurons and synapses, or perhaps fundamental particles, are endowed with the relevant powers to bring about the manifestation of apparently distinctive mental phenomena. If there are grounds to think they are sufficient, then the prospects for reduction look good. If not, then there is at least *prima facie* warrant for the emergentist to argue that a strongly emergent entity—such as *the mind*, considered as distinct from and novel with respect to, even if dependent on, the brain—ought to be posited which is endowed with the relevant causal power(s) to account for the behaviour or phenomena.

The final sections of this paper will attempt to show that the way this dialectic plays out is crucially sensitive to the way in which the relationship between powers and their manifestations is conceived—that is, to which of the accounts of the identity and individuation of powers outlined above is assumed.

### Examples of Emergence

When presented with a putative example of emergence and faced with the sort of dialectical situation outlined above, it will typically be the case that the available empirical evidence does not determine whether one ought to take an emergentist or reductionist attitude towards the case at hand. Why should this be the case? One reason is that empirical evidence is unlikely to ever *determine* that a case is a genuine case of emergence, for claims that a given phenomenon is emergent will always be defeasible: future evidence might show that some higher-level phenomenon which appeared to be emergent can in fact be fully accounted for by (previously undiscovered or not fully understood) features of the lower-level base phenomena upon which it depends. That this is the case implies that, at least in principle, the evidence might determine that one takes a reductionist attitude to a putative case of emergence. However, generally speaking, when some higher-level phenomenon is picked out as a putative example of genuine metaphysical emergence—such as the mind—compelling empirical evidence in favour of reductionism is absent: if such evidence were available, then the phenomenon in question would not have been selected as an example of emergence in the first place.

One might be tempted to think that the defeasible nature of any given putative case of emergence automatically favours reductionism—at least when coupled with some form of optimistic meta-induction that observes that many previous putative examples of emergence have been overturned and reasons that this trend will continue. This line of thought, however tempting, should be resisted. First, it is a highly-contentious matter as to just how many successful reductions have in fact been achieved and this contention thus carries over to the basis on which such an induction might be made. Second, putative examples of emergence, both currently and historically, are a highly heterogeneous group: chemical properties (e.g. Hendry 2010); the *elan vital* (introduced by Bergson, e.g. 1998 [1911]); folding behaviour in proteins (e.g. McCleish 2017); condensed matter phenomena such as the Fractional Quantum Hall effect (e.g. Lancaster and Pexton 2015); mental phenomena such as conscious experience (e.g. O’Connor and Jacobs 2003)... the list goes on. This heterogeneity means that over-arching inductions such as the one sketched above are likely to be inappropriate unless relevant similarity between cases can be demonstrated: that the *elan vital* has been explained away, say, may have little relevance to the question of whether conscious experience is a strongly emergent phenomenon. For similar reasons, it is unlikely that any general inductive argument could be run in the opposite direction, favouring emergentism.

In the absence of determining empirical evidence or general arguments, whether one should take a putative example of strong emergence seriously or not will be a matter of balancing various considerations to decide between the competing views. The next section will address one sort of consideration that will play a key role: the degree to which the putative example of emergence strikes one as genuinely surprising, novel and distinctive with respect to the lower-level phenomena from which it is claimed to emerge.

### Multi-tracking: A Worry for Emergentism

Consider first the multi-track mutual manifestation and multi-track stimulus manifestation views. According to these accounts of powers (especially those versions which endorse *qualitative* multi-tracking), what manifestation will issue from the operation of a power on a given occasion is highly context sensitive and will not be determined by the power alone.

If the multi-track mutual manifestation view is correct, then any given power is directed towards a wide range of different manifestations, and which manifestation occurs on a given occasion will be determined by which mutual manifestation partners the power interacts with on that occasion. Thus, it is built into this conception of powers that the very same powers will manifest differently—perhaps radically differently—in different complex situations. On the multi-track stimulus manifestation view, powers have a range of manifestations, and which occurs on a given occasion will again be highly context sensitive, conditioned not on other powers with which the power works together, but rather on what particular stimulus triggers the operation of the power on the relevant occasion. Again, it should be expected on this view that the very same power manifests differently in different complex situations, just as with the account discussed above.

Thus on either of these views it should be expected that in different complex situations diverse manifestations will occur, even given relatively few basic powers. Certain manifestations may not occur in any situation except for one with some highly-specific make up. How does this impact upon the debate between emergentists and reductionists? In a candidate example of emergence, the impetus to posit genuinely novel and distinct entities to account for some idiosyncratic behaviour which occurs only when certain complex situations are realised is dependent on the notion that the powers or properties of the base entities are not sufficient to account for or produce that behaviour—either alone or in combination. But multi-track views call this notion into question: this account of powers allows that the very same powers can do wildly different things in different circumstances.

This provides a framework for a reductionist response to any candidate example of emergence. Whilst the reductionist may concede that candidate examples of strong emergence involve behaviour which only occurs when certain complex situations are realised and which could not be predicted from knowledge of how the components of such situations behave in other context; they can resist the claim that this epistemic gap warrants an ontological bridge. Rather, they can situate the novelty and distinctness that the strong emergentist locates in distinctive higher-level objects or properties in the multi-track powers of the base entities themselves: the realistation of certain complex situations brings together partnerings of powers, or allows for specific stimuli, which could not occur outside of those situations, and this accounts for the powers of the base entities making manifest behaviour which *could not* occur otherwise—manifestations towards which the powers of the base entities were directed to all along, but which could only be made manifest in specific, complex circumstances. Thus, apparent examples of emergent phenomena are just that: appearances. There may be epistemic emergence, but there is no need to posit novel properties or powers to account for novel behaviour, and therefore candidate examples of emergence do not involve any genuine ontological novelty.

It remains for the reductionist to provide positive reasons for thinking that this ontologically deflationary account of apparently emergent phenomena is preferable to strong emergentism. Without such reasons, the emergentist might complain that at best multi-tracking offers a *possible* reductionist alternative, but the potential for an alternative account doesn’t favour one account or the other: at best, the debate might have arrived at a stalemate. There are at least two ways in which the reductionist might provide positive reasons. The first would involve outlining a plausible mechanism by which powers might combine such that they would produce certain kinds of novel behaviour only in certain specific circumstances. Whilst there isn’t space in this paper to address this issue in detail, there have been attempts in the powers literature to outline just such a mechanism, in particular through discussions of non-additive and non-linear composition rules for powers (e.g. Mumford and Anjum 2011, ch. 4).

In combination with the first reason, the reductionist might note that once one has both an account of powers in place which accommodates novel behaviour without genuine ontological novelty, and one has outlined a plausible mechanism by which the powers of base entities might produce novel manifestations in complex situations (in the form, say, of non-additive and non-linear composition rules), then theory choice considerations favour the reductionist account, and so there is no stalemate situation. The proposed reductionist account will have advantages over the emergentist alternative in terms of parsimony: it posits fewer entities, of fewer distinct kinds, to explain the same phenomena. It might also be thought to be more unificatory than the emergentist alternative: as noted earlier, candidate examples of emergence are highly heterogeneous, and where the emergentist will need to posit a distinct ontological regime to account for each, the reductionist appealing to multi-track powers of base entities will subsume all cases under a single explanatory framework. Thus, there are positive reasons for supporting the reductionist account once a multi-track account of powers is adopted.^3^

Whilst multi-tracking views may not strictly entail the falsity of emergentism, they seriously undercut the warrant for appealing to genuine ontological novelty to account for novel behaviour: that novel manifestations occur in such situations is to be expected, and so one needn’t posit exotic emergent higher-level phenomena to explain their occurrence. One way of thinking about this problem is as a specific version of the ‘collapse objection’ which holds that apparently emergent entities will, on closer inspection, collapse to lower-level entities (e.g. Taylor 2015). Thus, it seems that if this is the view of powers assumed, then it becomes very hard to see what sort of evidence the emergentist could bring forward to support their case, and the debate is prejudiced in favour of reductionism.

### A Problem Concerning Single-Track Partner Powers

According to the single-track mutual manifestation view, for a power to produce its manifestation it needs to work in concert with one or more specific partners which are also directed towards producing just that manifestation with just those partners. Remember, on this view, as each power is directed towards a single manifestation type, the candidate example of emergence being considered cannot be explained away by appeal to the complexity of the situation, as discussed above: in order to account for the novel behaviour or phenomena, no power directed towards any other manifestation—so no power of the entities involved in that situation that manifest otherwise—can be called upon, for then these would be multi-track powers. It seems then, that if this view were correct, then there would be warrant for supposing that some genuinely novel powers are operating to bring about the manifestation which constitutes the candidate example of emergence. This account allows for, but doesn’t entail, strong emergence in a way that the two accounts previously discussed do not.

But there is an interesting, and perhaps uncomfortable, consequence of this view for emergentists. What the view requires is that for the emergent power to manifest, there must be some partner or partners present for it to do so—partner(s) directed toward producing the very same manifestation, and nothing else. The emergentist faces something of a dilemma: either the partner power is also emergent, or it belongs to the base entities. Each option has its drawbacks. If the partner is also emergent, then it seems that the ‘downwards’ causal force many emergentists want to ascribe to emergent entities is lacking. But if the partners are powers of the base entities, then it seems we must endow these entities with a whole host of powers directed towards manifestations with various potential emergent powers.

The problem here is not a general one concerning partnering between different ‘levels’, nor is it a problem concerning familiar issues such causal closure. Rather, the worry is this. If:

(i) powers are single-track—that is, directed to only a single manifestation
(ii) powers operate according to the mutual manifestation model—that is, they require partner powers to manifest
(iii) emergent powers partner with powers of the base entities

Then the base entities will have to be endowed with ‘bespoke’ partner powers for every kind of emergent power. Suppose that some set of physical fundamentals the Bs constitute the base entities. And suppose that there is relatively widespread strong emergence: when the Bs are in the right arrangements, there are emergent chemical powers, biological powers, mental powers (and so on). Then, for these emergent powers to have partners (and given the singly-track mutual manifestation model, remember each power must have it’s own specific partner(s), as discussed in Sect. 4.3—if partners could do ‘double duty’, then they’d be multi-track), the base entities the Bs must be endowed with specific partners-for-the-chemical-powers, partners-for-the-biological-powers, partners-for-the-mental-powers and so on. And these will have to be distinct from the powers the Bs have which account for all their ‘B-level’ interactions.

Perhaps the emergentist could bite the bullet, and simply accept that this is how it is with emergent powers and their basal partners. The picture sketched above isn’t incoherent. It is, however, undesirable in several ways. First, it might strike some as overly costly: basal entities now need to be ascribed a whole host of additional powers. Second, there might be a concern that there’s an ad hoc element to this account: we start with the emergent powers and then basal partners get projected ‘down’ from the emergent level simply on the grounds that emergent powers are in need of them. Third, one might be concerned that this account threatens the sense of novelty that emergence typically requires: if the base entities are such that they have always had specific partner powers ‘ready to go’ for emergent powers, then it’s almost as if the emergent entity is somehow prefigured in the basal entities themselves.

Adopting the single-track mutual-manifestation account of powers presents, at the very least, a set of challenging problems for the emergentist. Maybe a sensible strong emergentist position could be developed which can deal with the worries raised above, but this is work yet to be done.

### Powers for Emergentists

This leaves the single-track stimulus manifestation account of powers, which appears to be the most felicitous for strong emergentists. On this view, each power is directed towards just one manifestation type, and so the presence of a novel manifestation in the form of idiosyncratic behaviour or phenomena in some complex situation gives warrant for positing a new power. And unlike the single-track mutual manifestation account, the positing of that new power doesn’t entail the positing of further powers in order to allow it to operate: on the single-track stimulus manifestation view the manifestation is brought about by the power alone, just so long as it is triggered by the relevant stimulus. As no further powers need to be posited there is no question about the status of these powers, and so it seems that the sorts of uncomfortable consequences discussed above will be avoided. One might think that an equivalent problem might arise concerning the stimulus for the power, however the view does not require that we posit some distinctive form of (emergent?) stimulus to trigger the emergent power, for it is consistent with this view that the very same stimuli can trigger different powers to produce different manifestations. Thus, worries concerning costliness, ad hoc-ery and so on do not appear to arise.

The foregoing has been something of a breakneck-speed exploration of how adopting different conceptions of powers can impact on how contentious examples of potentially emergent phenomena will be assessed. Far more could be said about each case, but I hope that enough has been done to render plausible a fairly modest conclusion: if one conceives of strong emergence in terms of the possession or bestowal of novel causal powers, then it is crucial to make explicit and to examine in detail how one’s conception of the nature of powers might influence one’s approach to the debate. Failure to recognise the importance of this issue risks parties to the debate talking past one another.

## Concluding Remarks

Are there emergent minds, possessed of distinctive, novel causal powers? Can conscious experience be reduced to neural activity or micro-physical goings-on? Nothing said in this paper implies any answer to these or related questions concerning the emergence or reduction of the mind. Rather, the principal concern of this paper has been to show that the way in which certain issues in the metaphysics of powers are resolved has ramifications for the debate between emergentists and reductionists, at least when strongly emergent entities are characterised as those entities which have or bestow distinctive causal powers relative to the basic entities from which they emerge. Relatively little attention has been paid thus far in the debate to the fact that there is no standard, universally accepted account of the nature of ‘causal powers’ (although see Baysan and Wilson 2017 for an excellent paper on related topics), and that this term can pick out entities with quite radically differing identity and individuation conditions depending on which account is assumed.

In particular, it has been argued that accepting certain accounts of causal powers can prejudice the debate, in some cases in favour of the reductionist, in others in favour of the emergentist. One conclusion that can be drawn from this is that in order for the debate to continue in good order, it is essential that these potential prejudices are made explicit and open for assessment. What has been provided here has only been a brief sketch of how these issues play out, so further work on the topic is called for.

## Footnotes

1 Why just in part? If the arguments put forward in this paper are correct, then the debate is also sensitive to distinctively metaphysical questions concerning the identity and individuation of powers, and these questions are not to be settled empirically. But empirical evidence will still be relevant for establishing candidate cases of emergence, and for assessing the plausibility of candidate reductions—see Robb (2018) for a recent discussion of this issue in the context of emergent mental causation.

2 In this paper I talk in the familiar terms of objects and their properties (remaining neutral on whether properties should be understood as universals or as particulars), which is suggestive of a two-category ontology. Alternative basic ontologies are of course available—for instance, if one favours a one-category trope ontology, then object-talk should be reconstrued in terms of bundles; instantiation-talk in terms of a bundling-relation (and of course this distinction does not exhaust the options: some opt for richer categorial structures including, for instance, events or kinds). I do not think anything said in this paper should be sensitive to these distinctions.

3 Shoemaker (2007, ch. 4) has suggested a similar response to emergentism that appeal to ‘micro-latent’ powers. Briefly, the idea is that novel behaviour could be accounted for in terms of the manifestations of (single-track) powers of the base entity which are only triggered in certain complex situations. This response, however, will have a much harder time meeting the challenge to provide positive support than the multi-track account outlined above. The account will not be more parsimonious than the emergentist alternative, as it must posit distinct powers to account for novel behaviour (it simply locates them at the lower, rather than higher, level). And the account will not be unifying—it will need to posit distinctive sets of powers for each candidate example of emergent phenomena.
